# Reanalysis on Performance of Microwave Phase Detector for Multisignals

**DOI:** 10.3390/s24248076

**Published:** 2024-12-18

**Authors:** Jing Deng, Hongxun Wang, Xin Xiang

**Affiliations:** Aviation Engineering School, Air Force Engineering University, Xi’an 710038, China; djin0662@126.com (J.D.); xxisdn2002@sina.com (X.X.)

**Keywords:** microwave phase detectors (MPDs), intermodulation effects, multiple signals, noisy electromagnetic environment

## Abstract

Microwave phase detectors (MPDs) are key components of instantaneous frequency measurement (IFM) receivers and phase interferometer direction finding (PIF-DF) receivers. In conventional analyses, there is seldom a major quantitative discussion of MPD characterization when multiple signals arrive at the same time, which is often the case in complex and noisy electromagnetic environments. We have reanalyzed the characteristics of MPDs with respect to filter effects acting on more than two RF signals and differential amplifiers, which are not considered in conventional analyses. First, a step-by-step mathematical model of the signal flow is developed, which creates a cross term between the two signals and naturally introduces intermodulation effects. Second, the new response characteristics of the MPD are evaluated by simulation. Finally, the intermodulation effects of zero-forcing and extreme-forcing were found simultaneously in the crosspoint frequency and near-frequency regions of multiple signals, which led to significant deviations and errors in the output of the MPD. This effect may have significant implications for IFM and PIF-DF receivers.

## 1. Introduction

With the increasing complexity of electromagnetic space, the efficiency of current receivers is facing more severe challenges. To better obtain the advantages of electromagnetic space and significant information advantages in noisy electromagnetic environments, it is necessary to strengthen reconnaissance and acquisition of signal frequency domain parameters. As an essential means of frequency measurement, instantaneous frequency measurement (IFM) and phase interferometer direction finding (PIF-DF) receivers can effectively measure the frequency characteristics of a single pulse. IFM receivers can improve interception probability for signal instantaneous frequency while ensuring a large instantaneous bandwidth. The phase interferometer direction-finding receivers can achieve real-time direction finding in multiple directions without guaranteeing high accuracy and anti-interference.

Microwave phase detector (MPD) is the core component of the IFM receiver and PIF-DF receiver, among others; its effectiveness and technical indicators become critical factors in the system [1]. The development of IFM is presented in article [2], where one of the limitations of IFM is mentioned, namely “simultaneous arrival signal indication”, which does not involve multisignal analysis. Another study [3] states that MPD cannot detect and encode errors in two RF signals if the frequency difference between the two signals is sufficiently large. When two RF signals overlap at the same time, the coding of the MPD output may not agree with the ambiguous data at any input frequency [4], but the principle of its erroneous frequency output is not discussed. Article [5] measured chirped and stable (simple) signals with three MPD-constructed instantaneous frequency measurement receivers and found that the outputs of the two signals near the frequency overlap can deviate significantly, but did not analyze the principle of its generation. In the current electromagnetic environment, the probability of signal overlap is extremely high, and the operational effectiveness of instantaneous frequency measurement receivers is limited when receiving overlapping signals [6], indicating that signal overlap has a significant impact on the response characteristics of MPDs. The existing literature focuses on improving the sensitivity, frequency accuracy, and frequency resolution and reducing the measurement error of instantaneous frequency measurement receivers from the technical level of hardware and algorithms [7,8,9,10,11,12,13,14], but it does not discuss the principle of signal overlap effect.

Hongxun Wang investigated the quantitative processing of double-pulse signals in the IFM system based on a phase discriminator [15,16], and Shiyan Wang investigated the quantitative processing of double-interleaved linear FM signals of IFM [17], but no more signals were analyzed in terms of their roles. Digital signal processing methods for frequency estimation have been proposed in [18], and the MUSIC algorithm [19], auto-regressive algorithm (AR) [20], Proony algorithm [21], Pisarenko algorithm [22] and improved Proony algorithm [5] have been used to estimate the frequency near the overlapping of the signals, but they have not analyzed the improvement of the coding based on the results of the principle of bias. Reference [23] investigates the principle of simultaneous arrival of multiple signals when the frequency difference between the signals is much larger than the instantaneous bandwidth, but it does not consider the interaction between the signals when the frequencies are similar. Although the multisignal case of IFM was addressed, only two signals were considered. Conventional IFM receivers cannot correctly respond to multiple signals at a particular moment; it is necessary to comprehensively assess the impact of multisignal overlap on traditional IFM receivers. However, as one of the essential means of frequency measurement, the conventional IFM receiver faces the problem of multi-signal overlap, which is the same problem as the interferometer direction-finding receiver, but research on core component MPD is also fundamental. Article [24] indicated that the frequency error near the signal overlap is large, and further research is needed to improve the performance of the receiver. In this paper, we quantitatively analyze and derive the formula based on the principle of MPD signal overlap error, and the theoretical results we deduce explain the principle of frequency abrupt change near the overlap of frequency points.

IFM receivers and PIF-DF receivers are often used in complex and noisy electromagnetic environments, and actual scenarios of MPD are inconsistent with the theoretical assumption of a single signal. Relevant conditions of the single signal cannot be applied to complex and changeable electromagnetic space, and frequency domain information measured by the IFM receiver cannot be wholly reflected in a single signal, so it is necessary to consider the interference of multiple signal emission sources and unknown signals comprehensively. Therefore, starting from the assumption of multiple signals, the response characteristics of no less than two RF signals acting on MPD were analyzed.

Hongxun Wang and others took the lead in studying the response characteristics of MPD to two signals, and they theoretically established a rigorous mathematical model and simulated and analyzed it but lacked understanding and research on the multisignal response characteristics of MPD [16,23]. Based on previous research, this paper examines the performance of MPD under the influence of multiple RF signals based on specific constraints and further studies the response characteristics of MPD in combination with complex electromagnetic space problems faced in practical engineering applications.

To help readers better understand the logical flow and content arrangement of this paper, the following is a brief overview of the structure of the paper. In the Introduction section, we focus on the research background of the paper as well as the current research status. In the Materials and Methods section, we depict the structure of the microwave discriminator and analyze the limitations of the response characteristics of the traditional microwave discriminator. Then, we combine the basis of the single-signal analysis principle with the assumption of multiple signals, fully consider the filter characteristics of the differential amplifier, and carry out an in-depth theoretical analysis of the response characteristics of not less than two RF signals acting on the microwave discriminator. Then, we analyze the response characteristics of the microwave phase discriminator with no less than two RF signals, and the Results section closely follows the theoretical analysis in the Materials and Methods section and verifies the theoretical derivation of this paper by combining the actual measurement results and several simulation results. Finally, in the Conclusions section, we summarize the work of this paper, not only pointing out the theoretical contributions of the research results to the field of microwave phase discriminators, but also looking forward to the broad prospects of their practical applications.

## 2. Materials and Methods

### 2.1. Traditional Analysis on the MPD

#### 2.1.1. Structure of the MPD

MPDs (also referred to as microwave interferometers) are components used to measure the phase difference between RF signals, which consist of two or more microwave transmission paths. MPDs are widely used in radar, communication, radio measurement, and other systems, with advantages including high sensitivity, high precision, high reliability, etc.

**Figure 1:**
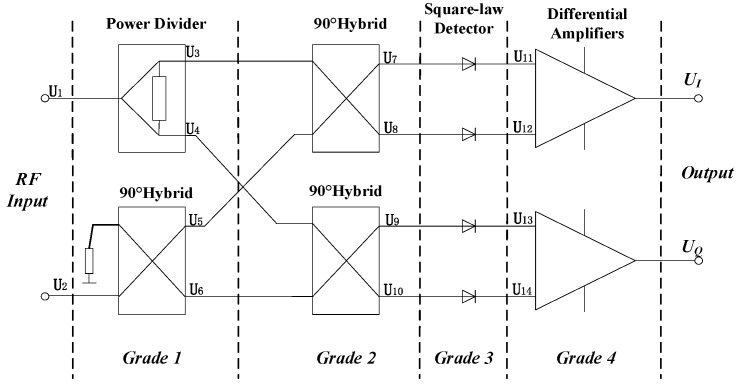
Structure of an MPD.

Figure 1 [16] shows the traditional structure of an MPD, composed of a 90° hybrid coupler, power dividers, square-law detectors, and differential amplifiers. First, two RF signals with differences in phase and frequency are fed. Second, four RF signals were produced and fed into a square-law detector. Third, four RF signals flow through the detector separately. Finally, a differential amplifier compares the phase difference between pairs of signals and converts this phase difference into a voltage proportional to it, or other easily measurable electrical signal parametric outputs.

#### 2.1.2. Response Characteristics of the Traditional MPD

For the MPD in Figure 1, RF signals at ports 1 and 2 were shown as follows:

(1) $$\left\{\begin{array}{c} U_{1}\left(t\right)=Ae^{j\left(\omega t+\varphi \right)}\;\;\;\;\\ U_{2}\left(t\right)=Ae^{j\left(\omega t+\varphi +\emptyset \right)} \end{array}\right.$$

(2) $$\left\{\begin{array}{c} U_{I}\left(t\right)=\frac{1}{2}KA^{2}\cos\emptyset \\ U_{Q}\left(t\right)=\frac{1}{2}KA^{2}\sin\emptyset \end{array}\right.$$

where *K* is the square-law detector detection coefficient and A is the amplitude of the input signal [23].

There was only one parameter: ∅, the phase difference between $U_{1}\left(t\right)$ and $U_{2}\left(t\right)$ of the MPD. Under this scenario, MPD outputs are shown in (2).

#### 2.1.3. Limitations of Traditional Analysis of the MPD

Equation (2) only applies to scenarios involving a single RF signal. The case of two signals was studied in reference [24]. The scenario of two RF signals was adopted in theoretical analyses, and MPD explanations are provided in references. However, the typical operation conditions of the MPD are complex and noisy electromagnetic environments, which differ from the theoretical scenario. Actual situations should be considered where more than two RF signals are act on the MPD.

### 2.2. Multisignal Paradigm and Analysis

The theoretical analysis idealizes the characteristics of the power divider, 90° hybrid coupler, power divider, square-law detector, and differential amplifier, which are not present in the actual MPD module. However, the idealized analysis can still be reasonably used for MPD studies. Based on the ideal example in Figure 1, the RF signal flow is modeled and divided into five sub-flows and six nodes: input, grade 1, grade 2, grade 3, grade 4, and output.

#### 2.2.1. Preconditions of Multiple Signal Inputs

N RF signals were assumed and fed into ports 1 and 2 of MPD in Figure 1, as shown in (3).

Complex and noisy electromagnetic environments were indicated in (3), where N RF signals were fed. In (3), $\varphi_{i}$ is the initial phase of each signal with frequency $\omega_{i}$, and $\emptyset_{i}$ is a difference in the phase of port two and port 1 for each signal.

(3) $$\left\{\begin{array}{c} U_{1}\left(t\right)=\sum_{i=1}^{N}A_{i}e^{j\left(\omega_{i}t+\varphi_{i}\right)}\;\;\;\;\;\\ U_{2}\left(t\right)=\sum_{i=1}^{N}A_{i}e^{j\left(\omega_{i}t+\varphi_{i}+\emptyset_{i}\right)} \end{array}\right.$$

#### 2.2.2. The Subprocess of Grade 1

In the first stage, the main working components were the power divider and 90° bridge. A power divider is a device that divides the energy of one input signal into two or more equal or unequal outputs. The 90° bridge has a function co-frequency combination, which can continuously sample transmission power along a certain direction of the transmission line and divide an input signal into two signals of equal amplitude and 90° phase difference.

One RF signal was divided into two signals of equal energy by the power divider with outputs of ports 3 and 4, shown in (4). Another RF signal was split into two signals of equal amplitude with a phase difference of π/2 by a 90° hybrid coupler; signal outputs of ports 5 and 6 are shown in (5).

(4) $$U_{3}\left(t\right)=U_{4}\left(t\right)=\frac{1}{\sqrt{2}}\sum_{i=1}^{N}A_{i}e^{j\left(\omega_{i}t+\varphi_{i}\right)}$$

(5) $$\left\{\begin{array}{c} U_{5}\left(t\right)=\frac{1}{\sqrt{2}}\sum_{i=1}^{N}A_{i}e^{j\left(\omega_{i}t+\varphi_{i}+\emptyset_{i}-\frac{\pi }{2}\right)}\;\;\\ U_{6}\left(t\right)=\frac{1}{\sqrt{2}}\sum_{i=1}^{N}A_{i}e^{j\left(\omega_{i}t+\varphi_{i}+\emptyset_{i}\right)}\;\;\;\;\;\;\; \end{array}\right.$$

#### 2.2.3. The Subprocess of Grade 2


(6) $$\left\{\begin{array}{l} U_{7}\left(t\right)=\frac{1}{2}\sum_{i=1}^{N}A_{i}e^{j\left(\omega_{i}t+\varphi_{i}\right)}+\frac{1}{2}\sum_{i=1}^{N}A_{i}e^{j\left(\omega_{i}t+\varphi_{i}+\emptyset_{i}-\pi \right)}\;\;\;\;\;\\ U_{8}\left(t\right)=\frac{1}{2}\sum_{i=1}^{N}A_{i}e^{j\left(\omega_{i}t+\varphi_{i}-\frac{\pi }{2}\right)}+\frac{1}{2}\sum_{i=1}^{N}A_{i}e^{j\left(\omega_{i}t+\varphi_{i}+\emptyset_{i}-\frac{\pi }{2}\right)}\\ U_{9}\left(t\right)=\frac{1}{2}\sum_{i=1}^{N}A_{i}e^{j\left(\omega_{i}t+\varphi_{i}\right)}+\frac{1}{2}\sum_{i=1}^{N}A_{i}e^{j\left(\omega_{i}t+\varphi_{i}+\emptyset_{i}-\frac{\pi }{2}\right)}\;\;\;\;\;\\ U_{10}\left(t\right)=\frac{1}{2}\sum_{i=1}^{N}A_{i}e^{j\left(\omega_{i}t+\varphi_{i}-\frac{\pi }{2}\right)}+\frac{1}{2}\sum_{i=1}^{N}A_{i}e^{j\left(\omega_{i}t+\varphi_{i}+\emptyset_{i}\right)}\;\;\; \end{array}\right.$$

The quadrature coupler (90° bridge) is the main working component in the secondary stage. Two 90° hybrid couplers were involved in grade 2 of the MPD. The RF signal flow of ports 3 and 5 was fed individually into a 90° hybrid coupler, and two signal flows were generated with each signal component of equal amplitude and 90° phase shift relative. The signal flows are shown in (6) at ports 7, 8, 9, and 10.

#### 2.2.4. The Subprocess of Grade 3

The four output signals of the above second stage after quadrature coupling are passed through the corresponding square law detectors to obtain the output signals of ports 11, 12, 13, and 14 as shown in (7) [16,23]. We initially chose the complex signal as the input signal for initial signal processing. However, it is necessary for us to convert it into a real signal for subsequent in-depth analysis and processing stages. In Equation (7), we use the detector property of the diode to extract the real part signal from the complex signal for subsequent processing and analysis.

(7) $$\left\{\begin{array}{c} U_{11}\left(t\right)=k_{1}{Re\left[U_{7}\left(t\right)\right]}^{2}\;\;\\ U_{12}\left(t\right)=k_{1}{Re\left[U_{8}\left(t\right)\right]}^{2}\;\;\\ U_{13}\left(t\right)=k_{1}{Re\left[U_{9}\left(t\right)\right]}^{2}\;\;\\ U_{14}\left(t\right)=k_{1}{Re\left[U_{10}\left(t\right)\right]}^{2} \end{array}\right.$$

where $k_{1}$ is the diode detection coefficient and the Re[ ] function represents the extraction of the real part from the complex signal.

#### 2.2.5. The Subprocess of Grade 4

Differential amplifiers were involved in grade 4 of MPD. The signals of the port $U_{I}$ were obtained by a differential amplifier with ports 11 and 12, and the signals of the port $U_{Q}$ were obtained in the same way with ports 13 and 14, shown in (8).

(8) $$\left\{\begin{array}{c} U_{I}\left(t\right)=k_{1}k_{2}f\left(\left|∆\omega \right|\right)\left[U_{12}\left(t\right)-U_{11}\left(t\right)\right]\\ U_{Q}\left(t\right)=k_{1}k_{2}f\left(\left|∆\omega \right|\right)\left[U_{14}\left(t\right)-U_{13}\left(t\right)\right] \end{array}\right.$$

where $k_{2}$ is the differential amplifier coefficient and $f\left(\left|∆\omega \right|\right)$ is the transfer function of the differential amplifier.

Two critical improvements in (8) were not considered in the traditional analysis of MPD. The first was the scenario of multiple RF signals involved in the abovementioned preconditions. The second were the frequency characteristics of the differential amplifier $f\left(\left|∆\omega \right|\right)$, which were usually used as low-pass filters. The results of $U_{I}$ and $U_{Q}$ ports can be merged using (7) and (8), as shown in (9).

(9) $$\left\{\begin{array}{c} U_{I}\left(t\right)=Kf\left(\left|\omega_{i}-\omega_{j}\right|\right)\left[{Re\left(U_{8}\left(t\right)\right)}^{2}-{Re\left(U_{7}\left(t\right)\right)}^{2}\right]\;\;\\ U_{Q}\left(t\right)=Kf\left(\left|\omega_{i}-\omega_{j}\right|\right)\left[{Re\left(U_{10}\left(t\right)\right)}^{2}-{Re\left(U_{9}\left(t\right)\right)}^{2}\right] \end{array}\right.$$

where $K=k_{1}k_{2}$.

When $\omega_{i}=\omega_{j}$, there was no crossover term between the signals, and they were independent of each other. In this case, $f\left(\left|∆\omega \right|\right)=f\left(\left|\omega_{i}-\omega_{j}\right|\right)=1$; the output result is shown in Equation (10).

(10) $$\left\{\begin{array}{c} U_{I}\left(t\right)=\frac{1}{2}K\sum_{i=1}^{N}{A_{i}}^{2}cos\emptyset_{i}\\ U_{Q}\left(t\right)=\frac{1}{2}K\sum_{i=1}^{N}{A_{i}}^{2}sin\emptyset_{i} \end{array}\right.$$

This is the traditional theoretical result of ignoring the intermodulation term. When $\omega_{i}\neq \omega_{j}$, there was a specific interference effect between signals when different signals were input simultaneously. The intermodulation term was the highlight of this paper, which is the reality of the situation.

(11) $$\left\{\begin{array}{c} U_{I}\left(t\right)=\frac{1}{2}K\sum_{i=1}^{N}{A_{i}}^{2}cos\emptyset_{i}+\frac{1}{2}K\sum_{i=1}^{N}\sum_{j=i+1}^{N}2A_{i}A_{j}f\left(\left|\omega_{i}-\omega_{j}\right|\right)cos\left[\frac{\left(\emptyset_{i}+\emptyset_{j}\right)}{2}\right]cos\left[\left(\omega_{i}-\omega_{j}\right)t+\left(\varphi_{i}-\varphi_{j}\right)-\frac{\left(\emptyset_{i}-\emptyset_{j}\right)}{2}\right]\\ U_{Q}\left(t\right)=\frac{1}{2}K\sum_{i=1}^{N}{A_{i}}^{2}cos\emptyset_{i}+\frac{1}{2}K\sum_{i=1}^{N}\sum_{j=i+1}^{N}2A_{i}A_{j}f\left(\left|\omega_{i}-\omega_{j}\right|\right)sin\left[\frac{\left(\emptyset_{i}+\emptyset_{j}\right)}{2}\right]cos\left[\left(\omega_{i}-\omega_{j}\right)t+\left(\varphi_{i}-\varphi_{j}\right)-\frac{\left(\emptyset_{i}-\emptyset_{j}\right)}{2}\right] \end{array}\right.$$

In this case, $f\left(\left|∆\omega \right|\right)=f(\left|\omega_{i}-\omega_{j}\right|)$. When the frequencies of the two are not very different, they affect each other. When two frequencies are very different, they do not affect each other; the output result is shown in Equation (11).

## 3. Results

The actual measurement results in [22,25,26] confirm a large frequency measurement error in the frequency measurement results at the frequency overlap of the two signals. To evaluate the above theoretical derivation results, we transmit two signals with overlapping frequencies through the USRP device and the Simulink function module in MATLABR2022b software, and the jump results of the measured frequency values at the frequency overlap are shown in Table 1.

As shown in Table 1, there is a large deviation in the frequency values at the signal overlap. To further evaluate the above theoretical derivation results, we use MATLAB software to verify the simulation experiments and compare the frequency measurement output results with or without the filter characteristics of the differential amplifier.

### 3.1. Preconditions of Scenario

**Figure 2:**
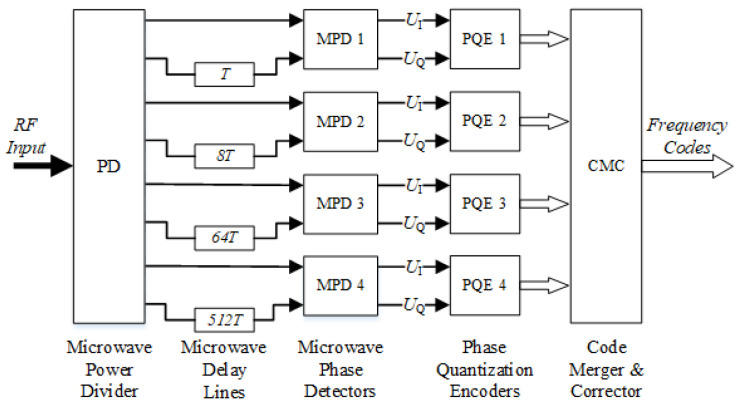
Four parallel microwave phase detector architecture.

The four-channel parallel microwave phase detector architecture is widely used in the fields of communication systems, radar systems, test and measurement, electronic countermeasures, and reconnaissance. This paper uses it as an example to study the influence of different delay lengths on the response characteristics of the MPD. The structure of the four-channel parallel microwave phase detector is shown in Figure 2.

In the following scenario, which is shown in Figure 2, an IFM receiver was adopted using a four-parallel channel architecture. Each channel had an MPD and a microwave delay line, which had different delay times relative to each other. A three-bit phase encoder was adopted for each channel, combined to indicate measured frequency values correspondingly.

As mentioned before, the filter characteristics of differential amplifiers were adopted, and the filter was assumed to be a two-order low-pass filter whose function was assumed to be (12).

(12) $$f\left(\left|\Delta \omega \right|\right)=H_{0}\frac{1}{{\left(\frac{\Delta \omega }{\omega_{p}}\pm \frac{1}{2q_{p}}\right)}^{2}+\left[1-{\left(\frac{1}{2q_{p}}\right)}^{2}\right]}$$

where $\omega_{p}$ is the pole frequency and $q_{p}$ is the quality factor. The two parameters were valued according to the practical applications. The following work is carried out in two cases of RF signal combination.

### 3.2. Fixed-Frequency and Linear FM Signals with Only One Frequency Crossing Point

**Figure 3:**
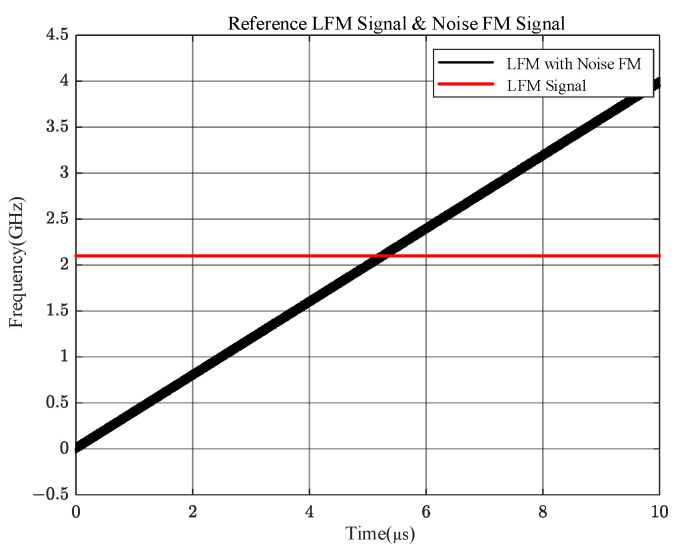
Input signals from two different frequency bands.

We performed simulation analysis with a multichannel IFM as an example to verify the results of the above theory. In this subsection, we analyze the output results of the four channels of the microwave phase detector when only one frequency is overlapped. Firstly, a signal with a stabilized frequency of 2.1 GHz is input, and the other is a 0~4 GHz LFM signal with a noise transient bandwidth of 100 MHz. The x-axis represents the simulation time, which lasts for 10 μs, and the y-axis represents the instantaneous frequency of the signal, which ranges from 0 to 4 GHz, as shown in Figure 3.

**Figure 4:**
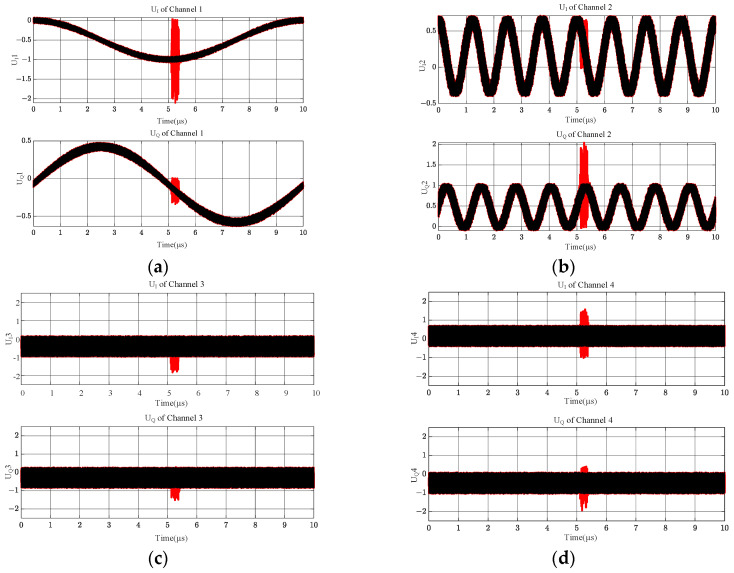
IQ values of 4 channels spanning from 0 to 4 GHz. (**a**) Outputs of IQ value in channel 1; (**b**) outputs of IQ value in channel 2; (**c**) outputs of IQ value in channel 3; (**d**) outputs of IQ value in channel 4.

Figure 4 shows the simulation results of the full-band IQ values of the above two different frequency band input signals obtained after the four microwave phase discriminators, in which the black curve is the output result of ignoring the overlapping term through Equation (10), and it can be seen that its value from beginning to end is very stable, without any abnormalities; the red color is the combination of this paper with the practical, and considering the overlapping term through Equation (11), it can be seen that when considering the overlapping term, the microwave phase discriminator $U_{I}\left(t\right)$ and $U_{Q}\left(t\right)$ will have huge, abrupt changes. The red color is the output result of Equation (11), from which it can be seen that considering the overlapping term, the output values of microwave phase discriminator $U_{I}\left(t\right)$ and $U_{Q}\left(t\right)$ will have a notable mutation near the overlapping of the two signals’ critical frequencies, and their values are closer to the values in the actual engineering.

The output values of $U_{I}\left(t\right)$ and $U_{Q}\left(t\right)$ through the shortest delay line, channel 1, of the MPD are shown in Figure 4a, output values through the sub-short delay line channel are shown in Figure 4b, and output values through the second-longest delay line channel are shown in Figure 4c. Output values through the most extended delay line channel are shown in Figure 4d. Significant biases were presented according to (11) at and near the cross-frequency from four channels in Figure 4. The biases were neither constant nor regular.

**Figure 5:**
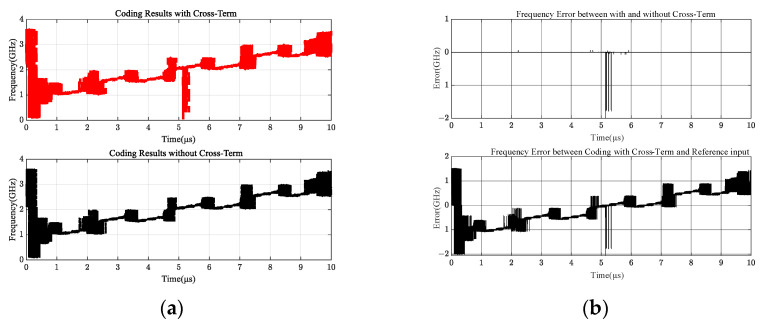
Comparison between encoding outputs. (**a**) Encoded output results; (**b**) coding deviation output results.

To improve the accuracy of measured values, the output results of four channels in Figure 4 that do not traditionally consider overlapping terms and output results given in this paper considering overlapping terms were quantified, and the output values of the phase identification module were encoded by three-bit encoding [27], as shown in Figure 5.

Figure 5a shows that the overlapping vicinity of the two signals dramatically influences the frequency measurement results when the intermodulation term is considered. More accurate coding bias results are shown in Figure 5b, which are consistent with the results of 5a, and the output result with or without intermodulation terms has a significant frequency deviation near the two overlapping signals. The coding deviation between the intermodulation term and the input is also the largest near the two overlapping signals. Therefore, the intermodulation term is a practical situation that cannot be ignored.

### 3.3. Fixed-Frequency and Two Linear FM Signals with Three-Frequency Crossings

**Figure 6:**
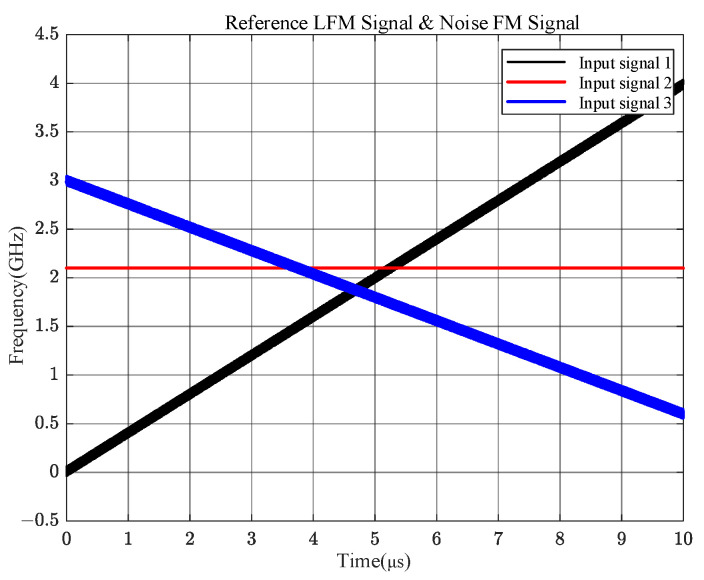
Input signals from three different frequency bands.

This subsection analyzes the output at the frequency overlap of three input signals. Based on the two input signals in case 2, an LFM signal was added with a negative growth frequency of 3~0 GHz and with a noise transient bandwidth of 100 MHz is input. The simulation results of the input signals are shown in Figure 6, where the three signals intersect at three different frequency points.

**Figure 7:**
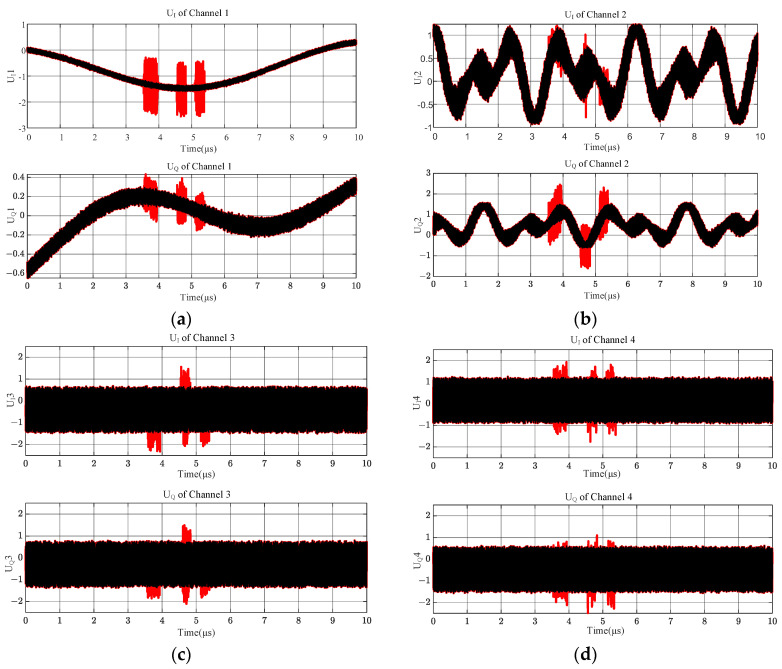
IQ values of 4 channels spanning from 0 to 4 GHz. (**a**) Outputs of IQ value in channel 1; (**b**) outputs of IQ value in channel 2; (**c**) outputs of IQ value in channel 3; (**d**) outputs of IQ value in channel 4.

Figure 7 presents a subplot of the coded output results for four different delay channels; the black curve is the output result without considering the differential filtering characteristics of the amplifier by Equation (10), and the red curve is the output result with considering the differential filtering characteristics of the amplifier by Equation (11). It can be seen that the red curve at the overlap of the three different frequency points of the frequency mutation is very large, more in line with the actual engineering measurement results. This indicates that the intermodulation term of the signal cannot be ignored in instantaneous frequency measurements.

**Figure 8:**
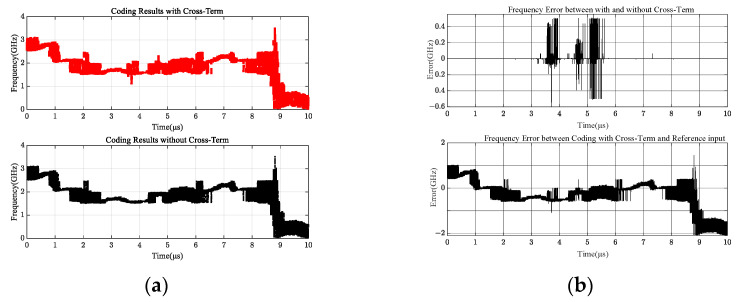
Comparison between encoding outputs. (**a**) Encoded output results; (**b**) coding deviation output results.

Figure 8a shows the actual measurements of the three different input signals with and without filter characteristics, and the difference between the two measurements is shown in Figure 8b. Combined with the input and output results of Figure 6, Figure 7 and Figure 8, it can be seen that in the range near the signal overlap, the frequency measurement does have a large error due to the response characteristics of the filter.

### 3.4. Frequency Crossings Cover the Entire Test Band

**Figure 9:**
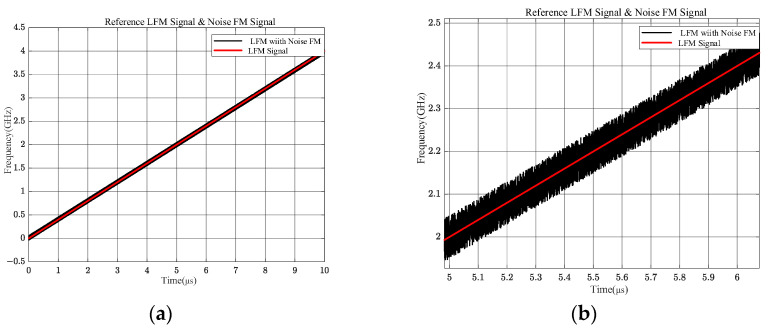
Two LFM signals with and without noises. (**a**) Panoramic view; (**b**) partial view.

In order to further verify the above theoretical speculations, the conditions of the two input signals were changed based on the above simulations, and further simulation analyses were performed. A 0~4 GHz LFM signal and a 0~4 GHz LFM signal with a noise transient bandwidth of 100 MHz were input and simulated within 10 μs. The simulation results of the input signals are shown in Figure 9. The black color represents the LFM signal disturbed by noise with instantaneous bandwidth of 100 MHz, and the red color represents the ideal LFM signal not disturbed by noise.

**Figure 10:**
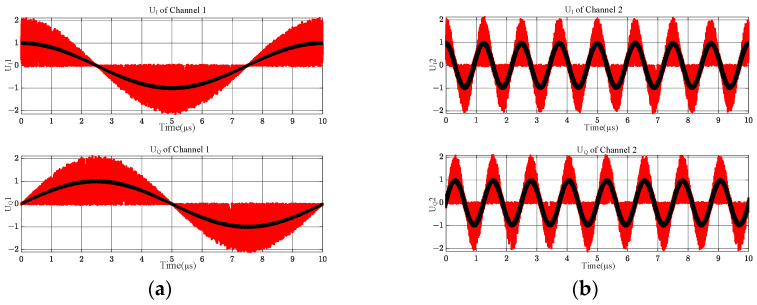

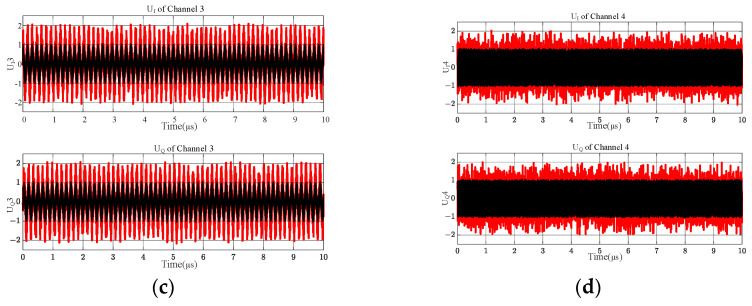
IQ values of 4 channels spanning from 0 to 4 GHz. (**a**) Outputs of IQ value in channel 1; (**b**) outputs of IQ value in channel 2; (**c**) outputs of IQ value in channel 3; (**d**) outputs of IQ value in channel 4.

A frequency crossover between the two signals in Figure 9 has always existed; Figure 9a shows its panorama, and Figure 9b shows some of the details. The theoretical analysis based on Equations (10) and (11) leads to the conclusion shown in Figure 10.

Figure 10a is shown as an output through the shortest delay line (channel 1), Figure 10b is output through the second shortest delay line (channel 2), Figure 10c is output through the second longest delay line (channel 3), and Figure 10d is output through the most extended delay line (channel 4). The red curves in the four subplots of Figure 10 result from the output measurements calculated using Equation (11), and the black curves result from the output measurements that do not take into account the intermodulation term using Equation (10).

The I and Q channel values of the MPD vary with the input RF signal. The intermodulation effect of the cross terms occurs only at and near the crossing frequencies of the two signals in Figure 4, while the intermodulation effect occurs near the overlapping of the three frequencies in Figure 7, and the intermodulation effect occurs throughout the entire frequency band in Figure 10. Based on the comparison of Figure 4, Figure 7 and Figure 10, this output is not accidental but inevitable.

**Figure 11:**
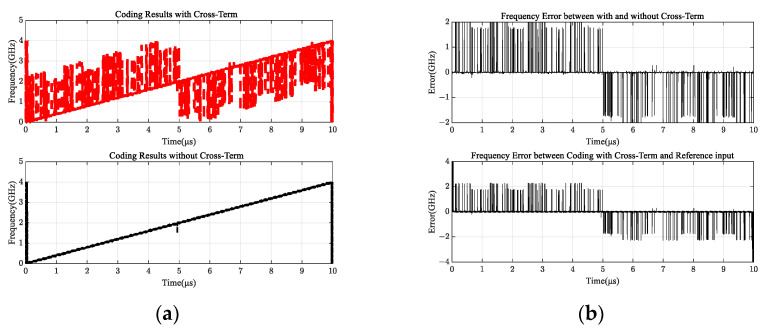
Comparison between encoding outputs. (**a**) Encoded output results; (**b**) coding deviation output results.

After quantifying four channels output in Figure 10, the output result of the phase identification module with three-bit encoding is shown in Figure 11. The encoding bias between the two cases is again compared in Figure 11; the upper subgraph shows the encoding output of overlapping items in this paper, and the lower subgraph shows that the production of overlapping items was not considered. Combined with a comparison of measurement results with or without intermodulation terms in the upper and lower subgraphs of Figure 11, the intermodulation terms significantly influence frequency measurement results, and even the phenomenon of zero and extreme forcing occurs. This shows that the influence of signal frequency overlap on the MPD cannot be ignored in complex electromagnetic environments, and subsequent research on MPDs should consider signal frequency intermodulation.

Figure 11 are divided into two subplots. Figure 11a shows the encoded output result, the upper subgraph shows the actual frequency measurement result with the intermodulation term in mind, and the lower subgraph shows the actual frequency measurement result without the intermodulation term. Combined with a comparison of the measurement results with and without the intermodulation term, the intermodulation term dramatically influences the frequency measurement results. Figure 11b shows the output result of the coding deviation, the upper subgraph shows the coding deviation between the output with and without the intermodulation term, and the lower subgraph shows the coding deviation between the output with the intermodulation term and the input. In summary, the influence of the overlapping frequencies of the two signals cannot be ignored.

## 4. Conclusions

Combined with the highly complex electromagnetic environment faced by the actual situation, based on the independent response characteristics of the MPD to two signals, the equation of N signal overlap was deduced from theoretical modeling. The working principle of multiple signals acting on the MPD was discovered. Secondly, combined with engineering practice, considering the filter characteristics of the differential amplifier, the influence of multisignal overlap on the MPD was simulated and analyzed. Finally, it is concluded that the near-frequency intermodulation effect may lead to a significant MPD measurement error. From the simulation results, it can be seen that input signals with similar frequencies will have a meaningful interaction and will also have a non-negligible impact on the response of the MPD. Therefore, when calculating output results, the intermodulation term is an essential item that cannot be ignored. Considering the influence of multiple signal sources and unknown interference signals in complex electromagnetic environments, the conclusions obtained from simulation results are of great significance for applying MPDs in practical engineering, especially in response characteristics of multiple signals.

## Figures and Tables

**Table 1:** The measured frequency result at the signal overlap.

Input Signal 1	Input Signal 2	Frequency Measurements at Signal Frequency Overlaps
2~4 GHz LFM signal	2.5 GHz stabilized signal	2.5 GHz→3.25 GHz
2~4 GHz LFM signal	2.7 GHz stabilized signal	2.7 GHz→3.62 GHz
2~4 GHz LFM signal	3.0 GHz stabilized signal	3.0 GHz→3.87 GHz
2~4 GHz LFM signal	3.5 GHz stabilized signal	3.5 GHz→4.63 GHz

## Data Availability

All data generated or analyzed in this study are included in the manuscript. All data in this study are available upon request from the corresponding author.
